# Highly Efficient Silicon Nanowire Surface Passivation by Bismuth Nano-Coating for Multifunctional Bi@SiNWs Heterostructures

**DOI:** 10.3390/nano10081434

**Published:** 2020-07-23

**Authors:** Mariem Naffeti, Pablo Aitor Postigo, Radhouane Chtourou, Mohamed Ali Zaïbi

**Affiliations:** 1 Laboratory of Nanomaterials and Systems for Renewable Energies (LaNSER), Research and Technology Center of Energy, Techno-Park Borj-Cedria, Bp 95, Hammam-Lif, Tunis 2050, Tunisia; radhouane.chtourou@crten.rnrt.tn (R.C.); ma_zaibi@yahoo.fr (M.A.Z.); 2 Instituto de Micro y Nanotecnología, IMN-CNM, CSIC (CEI UAM+CSIC) Isaac Newton, 8, E-28760 Tres Cantos, 28760 Madrid, Spain; pabloaitor.postigo@imn.cnm.csic.es; 3 Tunis University—National High School of Engineering of Tunis, 5 Avenue Taha Hussein, Tunis 1008, Tunisia

**Keywords:** Si nanowires, Bi nano-coating, surface passivation, visible-NIR emission, antireflection, minority carrier lifetime

## Abstract

A key requirement for the development of highly efficient silicon nanowires (SiNWs) for use in various kinds of cutting-edge applications is the outstanding passivation of their surfaces. In this vein, we report on a superior passivation of a SiNWs surface by bismuth nano-coating (BiNC) for the first time. A metal-assisted chemical etching technique was used to produce the SiNW arrays, while the BiNCs were anchored on the NWs through thermal evaporation. The systematic studies by Scanning Electron Microscopy (SEM), energy dispersive X-ray spectra (EDX), and Fourier Transform Infra-Red (FTIR) spectroscopies highlight the successful decoration of SiNWs by BiNC. The photoluminescence (PL) emission properties of the samples were studied in the visible and near-infrared (NIR) spectral range. Interestingly, nine-fold visible PL enhancement and NIR broadband emission were recorded for the Bi-modified SiNWs. To our best knowledge, this is the first observation of NIR luminescence from Bi-coated SiNWs (Bi@SiNWs), and thus sheds light on a new family of Bi-doped materials operating in the NIR and covering the important telecommunication wavelengths. Excellent anti-reflectance abilities of ~10% and 8% are observed for pure SiNWs and Bi@SiNWs, respectively, as compared to the Si wafer (50–90%). A large decrease in the recombination activities is also obtained from Bi@SiNWs heterostructures. The reasons behind the superior improvement of the Bi@SiNWs performance are discussed in detail. The findings demonstrate the effectiveness of Bi as a novel surface passivation coating, where Bi@SiNWs heterostructures are very promising and multifunctional for photovoltaics, optoelectronics, and telecommunications.

## 1. Introduction

Over the past few years, silicon nanowires (SiNWs) have aroused colossal attention worldwide both at the academic and technological level owing to the following fascinating peculiarities: (1) silicon is the second most earth-abundant material after oxygen, non-toxic, and environmentally friendly; (2) SiNWs have a distinct one-dimensional “1D” structure; (3) they have unique optical and electrical properties compared to bare silicon; (4) they have potential applications in several fields, ranging from solar cells, catalysis, and electronics to sensors [1,2,3,4,5]; and (5) they have affordable fabrication via numerous methods, including chemical vapor deposition (CVD), lithography, molecular beam epitaxy, laser ablation, and metal-assisted chemical etching (MACE) [3,4,5,6,7,8,9]. Among these methods, MACE is particularly intriguing and promising because of its simplicity, good cost-efficiency, and reliability [7,8]. It essentially consists of two processes—the formation of metal catalysts on the surface of Si substrates, and the subsequent etching leading to SiNWs formation—which can be implemented either in a single step (1-MACE) [3] or in two steps (2-MACE) [7,8,9]. Commonly, the MACE-grown SiNW arrays are covered with porous structures—namely, silicon nanocrystals (SiNCs)—resulting from the lateral etching of NW side walls [7,8,9]. Therefore, porous SiNWs are a promising host matrix to load and disperse a diverse set of functionalities and complexities according to the requirements.

Despite the aforementioned benefits, the porous SiNW surface often exhibits dangling bonds and defects generated during the fabrication process and the spontaneous oxidation in an ambient atmosphere. This leads to a deterioration of the SiNW performance and thus hampers their applications for commercialized products. To address these problems, one of the most effective strategies is SiNW surface passivation through loading metal nanoparticles (MNPs), resulting in dangling bond saturation and the formation of a stable passivation thick layer. In this context, many attempts at passivation have been reported in the literature, and several nanoscale metals have been anchored on SiNWs—e.g., Au; Ag; Pt; Pd; Cu; Li; and even bimetal NPs, e.g., Au-Ag, Au-Pd, and Pd-Ni [2,3,5,9,10,11,12,13,14].

The developed MNPs@SiNW hetero-nanostructure exhibits a remarkable enhanced performance as compared to the free metallic NP homo-nanostructure. For instance, photoluminescence (PL) enhancements with a factor of four and eight were recently reported for AgNPs-SiNW and AuNPs-SiNW heterostructures, respectively [10,15]. Amri et al. reported that the electrochemical deposition of Li on SiNW layers is responsible for the improvement of the minority carrier lifetime and the decrease in the reflectivity [13]. Moreover, Ghosh et al. demonstrate multifunctional AgNP-decorated SiNWs for sensing, photocatalysis, and light emission [9]. Silica NWs decorated with AgNPs and AuNPs are observed to offer absorption enhancement at the localized surface plasmon resonance wavelengths [5]. Other studies demonstrate that Au, Ag, Pt, Pd, Cu, Au-Pd, and Pd-Ni NP-coated SiNWs show enhanced photocatalytic properties and have been successfully used in the degradation of dyes and pollutants [2,3,9,11,12]. Besides this, Kim et al. not only demonstrated that nickel-oxide-decorated SiNWs with carbon shells (NiO@SiNW/C) exhibit significant electrocatalytic activity but that they also perform well as a counter electrode for dye-sensitized solar cells (DSSCs), with an excellent power conversion efficiency of 9.49% [16]. Other reports have highlighted the superior passivation of vertically aligned Si nanorods by a SiO_2_-multiwall carbon nanotube (SiO_2_/MWCNT) multishell [17]. Different techniques can be employed for the deposition of MNPs, including electroless deposition via a chemical solution [9,12,13], pulsed laser ablation [11], drop-casting [16], thermal evaporation [5,18], and more. The later technique is recommended and is one of the simplest and economical methods, enabling easy material anchoring with good properties and reduced surface damage [19,20,21].

Out of the expensive metals, Bismuth (Bi) is an effective alternative that has not been explored yet for the decoration of SiNWs toward enhanced performance. Thanks to its interesting properties, such as being environment-friendly with a low toxicity, being a good absorber, behaving as a smart optically active center in diverse host materials, and having a relatively low melting point, Bi-based materials are used in various applications, ranging from telecommunication, biomedicine, and white LEDs to lasers [22,23,24,25].

In this work, we report on the effective passivation of SiNWs via BiNC for the first time. The morphological and structural characterizations of the samples were studied, and then the visible-NIR (near-infrared) PL, reflectance, and minority carrier lifetime measurements were analyzed to examine the potential of Bi treatment. The developed Bi-coated SiNW (Bi@SiNW) heterostructure was analyzed to show that it is promising for applications in photovoltaics, optoelectronics, telecommunication, and other fields.

## 2. Materials and Methods

### 2.1. Reagents and Materials

The chemical reagents used in this work for the cleaning, etching process, and sample surface decoration, such as acetone, ethanol, isopropanol, hydrofluoric acid (HF, 40%), silver nitrate (AgNO_3_), hydrogen peroxide (H_2_O_2_, 35%), nitric acid (HNO_3_, 65%), and bismuth (Bi, 99%), were purchased from Sigma-Aldrich (Madrid, Spain). All of them were used without any purification. The single-side polished p-type silicon wafers were purchased from siltronics (Archamps, France), and the ultimately deionized water used for all the experiments was supplied by local sources.

### 2.2. Samples Preparation

#### 2.2.1. Fabrication of SiNWs

**Figure 1:**
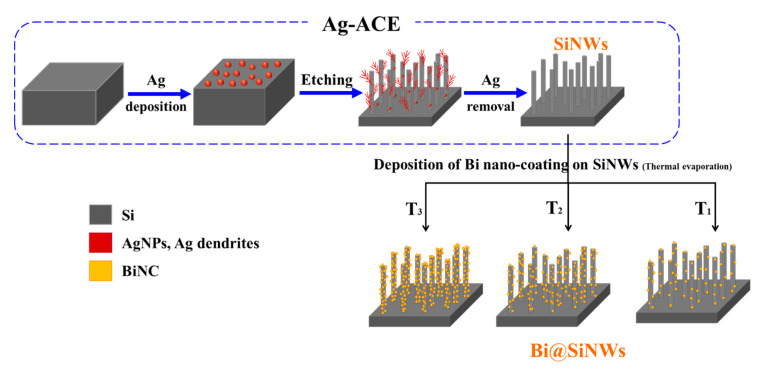
Schematic illustration of the fabrication process of the silicon nanowires (SiNW) and Bi-coated SiNW (Bi@SiNW) composite.

The SiNW arrays were synthesized by the Ag-assisted chemical etching (Ag-ACE) method of p-type silicon wafers (boron-doped; orientation: (100); resistivity: 1–20 Ω cm; and thickness: 500 µm). Figure 1 illustrates the fabrication process, which includes four steps as follows: (1) The silicon wafers were sequentially cleaned in acetone, ethanol, isopropanol, and deionized water (DI) in an ultrasonic bath for 15 min each. The cleaned wafers were then immersed in diluted HF for 3 min to remove the native oxide. (2) Silver nanoparticles were deposited onto the Si substrates using an aqueous solution composed of 4.8 M HF and 0.035 M AgNO_3_ for 1 min. (3) Si wafers covered with AgNPs were dipped into the etching solution of 4.8 M HF and 0.5 M H_2_O_2_ at room temperature for 20 min. (4) The resulting surfaces were rinsed with DI and then immersed in nitric acid for 15 min to remove the AgNPs and dendrites. Finally, the as-formed homogeneous black SiNWs were washed again with DI and dried with nitrogen. The SiNW fabrication mechanism is explained in our previous report [8].

#### 2.2.2. Deposition of Bi Nano-Coating on SiNWs

The Bi deposition on the as-prepared SiNWs was subsequently achieved through the thermal evaporation method. Initially, the chamber and tungsten boat were thoroughly cleaned by acetone. Then, high purity metal bismuth powder was placed in the cleaned tungsten boat and the chamber was evacuated to a high vacuum. At a vacuum of 10^−5^ torr, the Bi was heated indirectly by the heating of the tungsten boat, and the evaporation started to occur with a rate of 0.5 A·s^−1^. Different Bi thicknesses were deposited (T_1_ = 5 nm, T_2_ = 20 nm, and T_3_ = 35 nm) and were monitored via a quartz crystal microbalance. The obtained Bi@SiNWs nanocomposites were ultimately annealed at 100 °C for 15 min under a nitrogen atmosphere and labelled as “SiNWs + T_1_”, “SiNWs + T_2_”, and “SiNWs + T_3_” (Figure 1).

### 2.3. Characterizations

The morphologies of the samples were characterized using Scanning Electron Microscopy (SEM, FEI Varios 460, FEI Europe B.V., Eindhoven, The Netherlands). The energy dispersive X-ray spectra (EDX) were obtained by means of the SEM. The Fourier Transform Infra-Red (FTIR) spectra were taken using a Bruker IFS66v/s FTIR spectrometer (Bruker, Karlsruhe, Germany) with a resolution of 4 cm^−1^, where the mode was absorbance and the angle was normal. Photoluminescence (PL) spectroscopic analyses of the SiNWs and Bi@SiNWs were performed with a 405 nm laser wavelength, and all the measurements were conducted at room temperature. The reflectance measurements were performed via a Perkin Elmer Lambda 950 spectrophotometer (Perkin Elmer, Inc., Waltham, MA, USA). The microwave photoconductivity decay (µPCD) lifetime mapping was carried out with the use of a Semilab WT-2000-PVN machine (Semilab, Budapest, Hungary), which was equipped with a 905 nm laser excitation and a microwave source operating at ~10 GHz. The resulted effective lifetime maps show the sample-averaged lifetime.

## 3. Results and Discussion

### 3.1. Morphology and Structure

**Figure 2:**
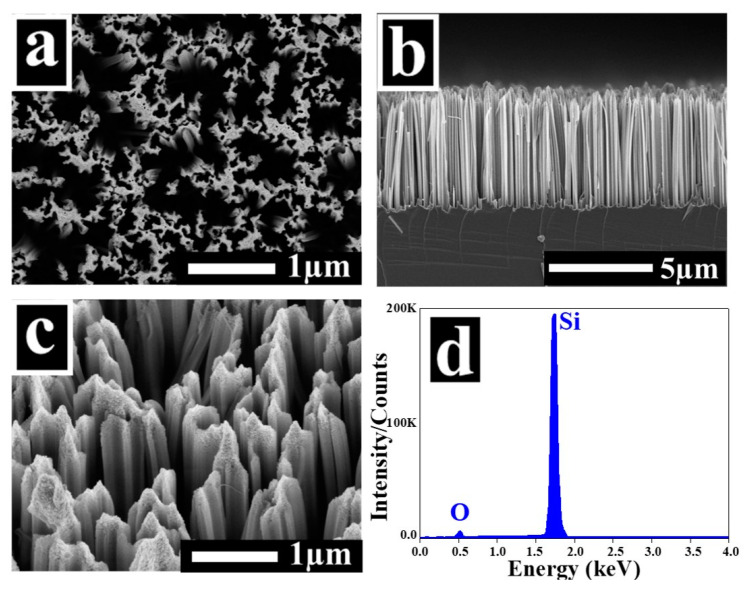
SEM images of as-prepared SiNWs: (**a**) top view, (**b**) cross-sectional view, and (**c**) 35° tilted view. (**d**) Corresponding energy dispersive X-ray spectra (EDX) spectra.

SiNW arrays were synthesized via a two-step Ag-assisted chemical etching method. An itemized explanation of the fabrication mechanism and the chemical reactions that occurred during the etching process are reported in our previous work [8]. Figure 2a depicts a typical top-view SEM image of the as-prepared SiNWs.

Forest-like SiNW arrays can be observed over the entire wafer. The NWs congregate together to form bundles, most likely due to van der Waals attraction [3,8]. The SEM cross-sectional view shown in Figure 2b indicates that the SiNWs are well aligned in the vertical direction following the orientation of the original silicon substrate, which is the (100) direction. The average length of the NWs is approximately 5 µm, whereas the diameters are in the range of 100 to 200 nm. From the 35°-tilted image, one can observe that SiNWs are covered with numerous porous structures called silicon nanoparticles (SiNPs), especially on the tips of the NWs. Figure S1 in the Supplementary Information further shows these SiNPs. These SiNPs are due to sidewall etching and cause quantum confinement (QC) effects, as discussed later in the PL study. An EDX analysis was carried out in order to figure out the elemental composition of the samples. Figure 2d shows the EDX spectra of the as-prepared SiNWs, where a strong signal from the silicon element is well distinguished with a small amount of oxygen. The detected oxygen is mainly due to the oxidation that happened during the etching of the silicon. Moreover, there are no detectable silver traces, indicating the complete removal of the AgNPs by nitric acid.

**Figure 3:**
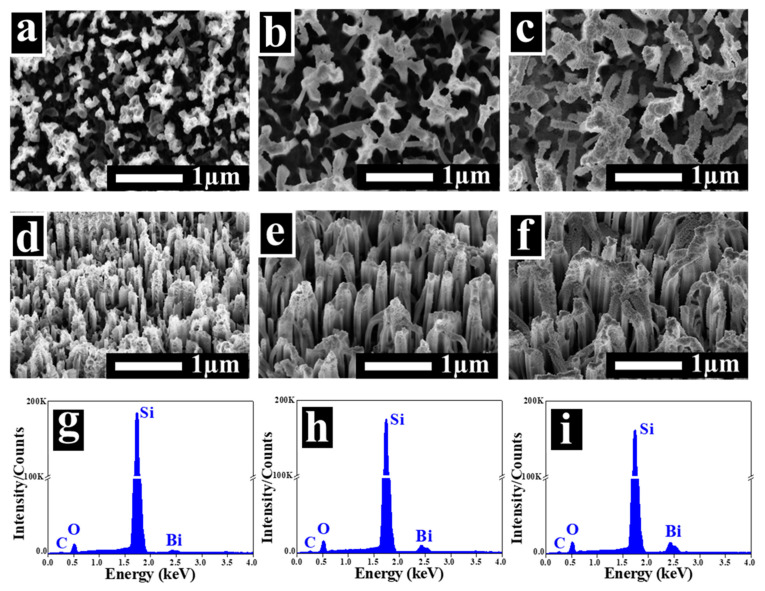
Top view, 35°-tilted view, and the corresponding EDX spectra of the decorated SiNWs, with Bi for (**a**,**d**,**g**) the T_1_ Bi thickness, (**b**,**e**,**h**) the T_2_ Bi thickness, and (**c**,**f**,**i**) the T_3_ Bi thickness.

Afterwards, the SiNW arrays were exposed to various amounts of Bi. Figure 3a–c illustrates the top view images of the Bi@SiNWs at different Bi thicknesses (T_1_, T_2_, and T_3_).

One clearly notices a discernible modification in the surface morphology of the SiNWs. The BiNC is observed to be uniformly distributed over the whole wafer regardless of its amount. The corresponding 35°-tilted view images are displayed in Figure 3d–f and show that the BiNC extends to the upper sides of the silicon walls, in addition to the tips. On the other hand, the Bi concentration was found to increase upon increasing the Bi loading. For the T_3_ Bi thickness, the particle density is much higher where they begin to agglomerate and partially fill the gaps between the bunches of SiNWs. The EDX elemental analysis of the Bi@SiNWs samples corroborates the presence of Bi (Figure 3g–i). By increasing the Bi amount (from T_1_ to T_3_), the signal intensity increased for Bi, whereas it decreased for Si, which is consistent with an increase in the Bi thicknesses. One can note that there are no discernible impurities other than a tiny quantity of carbon barely detectable, which could possibly have arisen from surface contamination during the Bi evaporation process. This is compatible with the FTIR results, as discussed later. One may deduce that the Bi@SiNW nanocomposite is successfully synthesized.

### 3.2. FTIR Analysis

**Figure 4:**
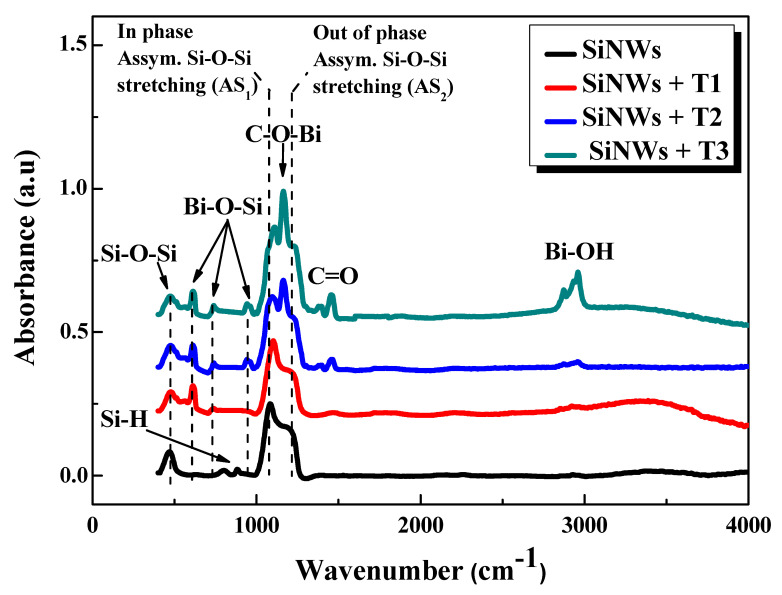
FTIR absorption spectra of the untreated SiNWs and the Bi-treated SiNWs at different thicknesses (T_1_, T_2_, and T_3_).

The modifications of the SiNW surface composition after the deposition of Bi were investigated using FTIR spectroscopy. The analysis was performed on the untreated SiNWs sample as background and Bi-modified SiNWs at different thicknesses (T_1_, T_2_, and T_3_). The FTIR spectra were taken in the absorption mode in the 400–4000 cm^−1^ spectral range and are depicted in Figure 4.

The characteristic asymmetric stretching vibrations (AS) of Si–O–Si are recorded in all the spectra and appear in the region between 1000 and 1300 cm^−1^. This signal includes a strong band at 1080 cm^−1^ related to the AS_1_ vibration mode and a shoulder at 1200 cm^−1^, attributed to the AS_2_ vibration mode. The AS_1_ outcomes from the motion of the adjacent oxygen atoms move in phase with each other, whereas at AS_2_ the oxygen atoms move 180° out of phase. The presence of this shoulder is a feature of the IR spectra of the SiO*_x_* material, where the x value is equal to one or higher [8,26,27]. Around 465 cm^−1^, an Si−O−Si vibration is also observed [8,26]. For the Bi-free SiNW sample, Si–H bending vibrations are located around 875 cm^−1^ [8,27].

On the other hand, it is easy to notice the appearance of new vibration peaks after the Bi deposition. In the literature, many authors have reported that metal-oxygen vibrations modes occur in the range of the spectrum from 400 to 700 cm^−1^ [28,29,30], while other works have reported that these latter vibrations appear below 1000 cm^−1^ [31,32]. Hence, the new peaks situated at 614, 733, and 940 cm^−1^ can be attributed to the formation of new bismuth–oxygen bands. The peaks at 1170 and 1460 cm^−1^ were assigned to the C–O–Bi and C=O vibrations, respectively [28,29]. The band at around 3000 cm^−1^ is attributed to the Bi–OH vibration [28]. We notice that the spectra of the Bi-treated samples are closely similar to an increase in the absorbance peak intensity versus the increasing Bi thickness; this is consistent with the increment of the Bi amount, as seen above in the SEM images.

### 3.3. Photoluminescence Spectroscopic Analysis

The aim of the PL study is to examine the impact of nanoscale dimension of Bi particles on the visible and NIR PL emission of the MACE-grown SiNWs. All the PL spectral measurements of the different samples were recorded at room temperature and under identical conditions.

#### 3.3.1. Visible PL

**Figure 5:**
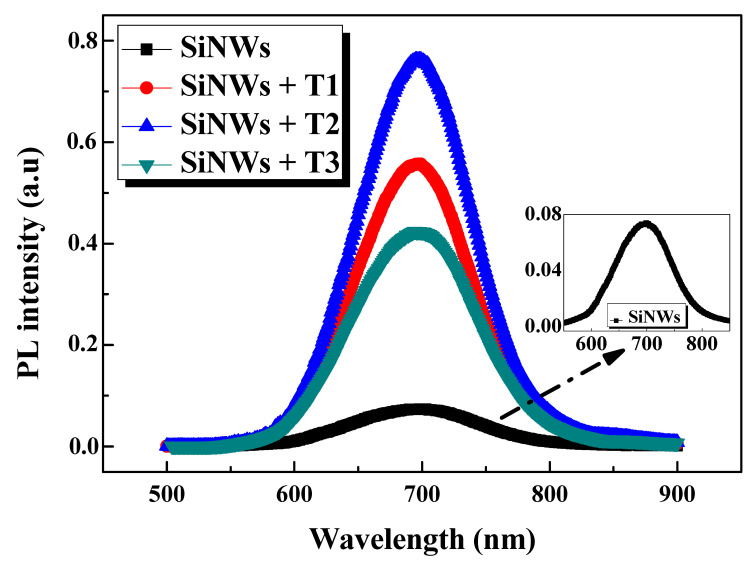
Photoluminescence (PL) spectra of Bi-free and Bi-modified SiNW samples at different Bi thicknesses (T_1_, T_2_, and T_3_).

The comparison of the PL spectra of the mesoporous SiNWs before and after the deposition with Bi at different thicknesses of Bi (T_1_, T_2_, and T_3_) are shown in Figure 5 over the wavelength range of 500–900 nm.

Interestingly, all the Bi-treated samples present an enormous enhancement in the PL intensity with respect to that of bare SiNWs. Furthermore, all the observed Gaussian PL bands are centered at 695 nm (1.78 eV), without record of any considerable shift, though the surfaces are modified by BiNC, suggesting the successful passivation of the samples [31].

As seen from the inset of Figure 5, even the as-grown SiNWs without the Bi coating exhibit visible PL emission, regardless of its extent. This emission could be ascribed to various reasons, including: (1) quantum confinement (QC) effects; (2) SiO*_x_*/Si interface defects and/or defect states in the oxide surface related to the Si–O bonds; (3) the radiative recombination of excitons in the small self-grown Si nanocrystals (SiNCs) present in the nanowire sidewalls, in terms of the QC model [7,8,33,34]. However, the latter explanation remains the most approved one, and the SiNCs size could be determined from the spectral position of the PL peak using the equation described below [34]:

(1) $$E=E_{g}+\frac{3.73}{d^{1.39}},$$

where E is the PL peak position (eV), E_g_ is the band gap of C–Si (1.12 eV), and d is the SiNC size (nm).

The broad emission may be due to the large size distribution of the SiNCs along the mesoporous SiNWs [7]. However, this PL emission of the as-grown SiNWs is unstable owing to the spontaneous oxidation in an ambient atmosphere, which provides recombination centers and thus reduces the efficiency of its optical properties. Therefore, the SiNW surfaces were modified by BiNC deposition to achieve a well-passivated nanocomposite. Effectively, a drastic improvement of the PL intensity is observed after the Bi coating is applied to the mesoporous SiNWs. The integrated PL intensities of all the Bi@SiNWs samples were found to increase until reaching about nine-fold enhancement, as presented in Table 1.

Several factors might be responsible for the enhancement of the PL intensity after Bi deposition. These are: (1) The removal/decrease of the interfering agents which hamper the radiative recombination of the photoinduced electron–hole pairs. (2) Bi treatment leads to a reduction in the deep trap density and quenches a large part of the silicon dangling bands generated during the etching process for SiNW formation. Thus, a passivation of the non-radiative centers occurs via the substitution of the unstable Si–H bands by the stable Bi–O–Si bands, Bi–OH, and other carbon-based functional groups, as revealed from the FTIR analysis [13]. (3) A possible increase in the density and/or creation of new radiative centers under the photo excitation of the charge carrier generated from the Bi deposits. (4) Plasmonic enhancement via BiNC [7].

At low Bi thicknesses (T_1_), the BiNC exists in islands and/or isolated clusters, leaving enough dangling bands to induce a relative lack of surface passivation. The highest enhancement factor of PL intensity is recorded for a moderate thickness (T_2_) of around nine-fold. When exceeding this latter optimum thickness, we observe a dwindling of the enhancement factor, but it remains much more intense than that of the as-grown SiNWs (around five-fold). This may be due to the consumption of the available silicon to form the alloy and also to the growth of large nanoclusters of Bi that ultimately aggregate together, forming a relatively thick Bi layer, and thus the light reaching the active SiNWs/NCs core is reduced. Another reason is that the large amount of Bi may destroy part of the radiative centers. Therefore, the PL emission intensity of the Bi@SiNWs is sensitive to the amount of Bi deposits, and judicious optimization is generally required to obtain a high passivation quality.

Generally, SiNWs passivation by metal deposition has a beneficial impact on optical proprieties. Enhancements with a factor of four and eight were recently reported for a SiNW/AgNP composite and a SiNW/AuNP composite, respectively [10,15]. Amri et al. show that the passivation of SiNWs with lithium not only improves the luminescence but also ensure a long PL lifetime. [13]. Herein, the Bi coating on SiNWs achieves an interesting PL enhancement, reaching a factor of nine-fold, as seen above, as well as the stabilization of the NW structure, which is very interesting and promising for nanoscale optoelectronic devices.

#### 3.3.2. NIR PL

**Figure 6:**
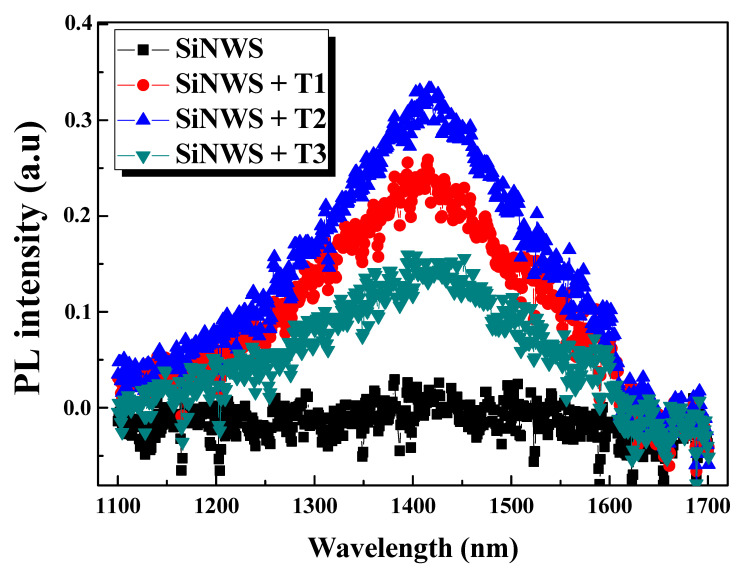
Near-infrared (NIR) PL spectra of Bi-free and Bi-modified SiNW samples at different Bi thicknesses (T_1_, T_2_, and T_3_).

Figure 6 displays the measured near-infrared PL spectra of Bi-free and Bi-modified SiNW samples. Besides the visible PL, we can detect a broad NIR PL emission from the Bi@SiNW samples, peaking at 1400 nm and ranging from 1100 to 1700 nm, covering the entire telecommunication window.

A similar NIR PL emission was recently reported for bismuth-based different host materials, such as glasses, oxides, crystals, thin films, zeolites, and others [22,23,35,36,37,38,39,40,41]. To our best knowledge, this is the first observation of NIR luminescence from Bi-coated SiNWs. This not only gives rise to a new family of Bi-doped materials operating in the NIR, but is also very effective in terms of emission bandwidth, spanning the whole NIR region. We strongly anticipate that this developed material system will be very interesting and promising as an optical amplifier at optical telecommunication wavelengths.

On the other hand, no apparent NIR signal is detected for pure SiNWs, suggesting that the NIR emission in the Bi@SiNWs samples is assigned to Bi NIR active centers. This is dissimilar to one earlier report, where the NIR PL is obtained from SiNWs and ascribed to the phonon-assisted radiative recombination of electron–hole plasma as well as to the SiO*_x_* layer [7]. Different kinds of Bi centers, including Bi^0^, Bi^+^, Bi^2+^, Bi^3+^, and Bi clusters [22,37,38,39,40,41], may exist, resulting from when Bi melts during the evaporation process. However, based on earlier studies, only bismuth with low valence states (Bi^0^, Bi^+^) or bismuth clusters originate the NIR emission, as they are the NIR-active Bi centers, regardless of the starting host material [36,37,38,39,40,41]. It is noteworthy that the spectral shapes and position of the maximum are identical for all the Bi@SiNWs samples. Besides, one can note that the changing trend of the NIR emission intensity of the samples is the same as the visible emission, where the highest PL intensity is recorded for the T_2_-moderated Bi thickness. However, visible PL emission is stronger than the NIR one, which may be due to the additive contribution of SiNW emission.

In short, both greatly enhanced visible PL and effective NIR emission occurred via the Bi@SiNW composite, which shed light on a new family of optically active materials and will be a starting point for future research on such composites. All the aforementioned peculiarities make the Bi@SiNW composite very promising and multifunctional in various applications, from light-emitting diodes and photonic devices to telecommunications.

### 3.4. Reflectance Analysis

**Figure 7:**
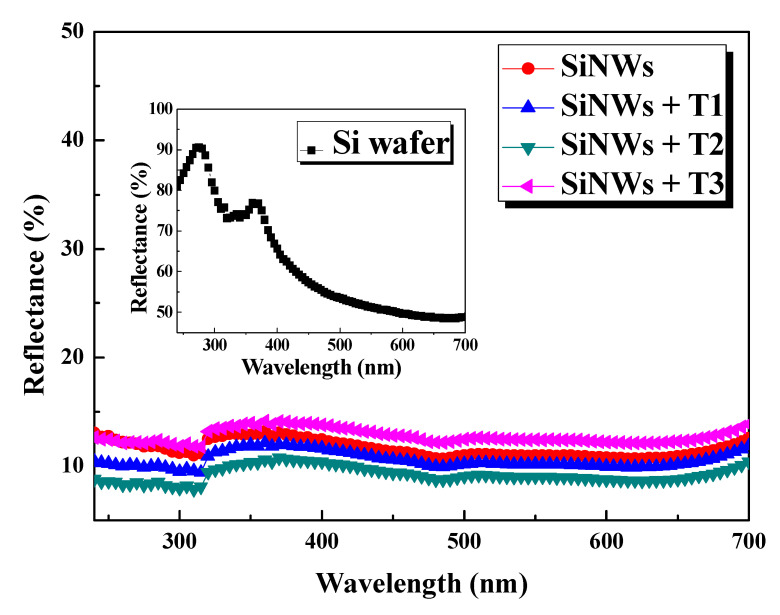
Reflectance spectra of Bi-free and Bi-modified SiNWs samples at different Bi thicknesses (T_1_, T_2_, and T_3_). The inset displays the reflectance spectra of the untreated Si substrate.

Figure 7 shows a comparison of the reflectance of the as-grown SiNWs, Bi@SiNWs at different thicknesses of Bi (T_1_, T_2_, and T_3_) and the corresponding bulk silicon wafer. The measurements were performed over the wavelength range of 250–700 nm, covering the major spectral irradiance of sunlight, which is vital for Si solar cells.

**Figure 8:**
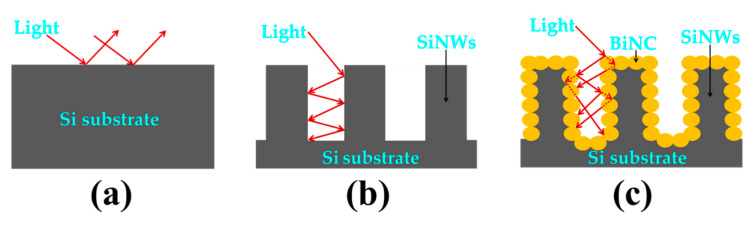
Schematic illustration of optical path and reflections occurring at (**a**) the untreated planar Si substrate, (**b**) the SiNWs, and (**c**) the Bi-modified SiNW structure.

The average reflectance of the untreated planar Si substrate is about 50–90% in the UV-visible range, as shown in the inset of Figure 7. These relatively high values are due to the smooth silicon surface and the absence of incident light-trapping structures (Figure 8a). The peaks at 274 and 367, as observed in the graph of Si wafer, arise from the inter-band transitions of Si [41,42]. After the formation of vertical SiNWs, a strong suppression in the reflectance to about 10% throughout the entire wavelength range occurred, which is consistent with the black surface appearance against the grey and polished Si substrate. This sharp drop in the reflectance is ascribed to the gradient of the refraction index and to the tapering nanowires structure [8,13,43].

As a matter of fact, this structure leads to light trapping, owing to the multiple reflections back and forth in the inner surface of SiNWs, as elucidated via the schematic illustration of the optical path in Figure 8b.

Further improvement of the antireflection performance occurs after the deposition of Bi on the SiNW arrays, where the average reflection reaches 8%. This can be due to the following reasons: (i) the BiNC causes higher absorption due to the surface plasmon resonance effect [7] and (ii) enhances the textured surface and the optical path by providing additional light fluctuations (Figure 8c), which increase the capture ratio of photons.

This result exhibits that Bi is an efficient material, not only in preserving the optical properties of SiNWs, but also it could be considered as an antireflection coating. However, as readily seen in Figure 7, the reflectance scantily increased for the T_3_-Bi thickness. We correlate this observation to the consumption of the available silicon to form the alloy, as well as to the growth of large nanoclusters of Bi that ultimately aggregate together, forming a relatively thick Bi layer.

Thus, the void between the wires decreases, leading to a reduction in the light reaching the active SiNWs and then a reduction in the light trapping phenomena. Therefore, the judicious optimization of the amount of Bi coating is essential.

Both SiNWs and Bi@SiNWs demonstrate excellent antireflection behavior, which is extremely promising for developing high efficiency solar cells and may shed light on Bi as a new antireflection layer coating.

### 3.5. Minority Carrier Lifetime Variation

It is quite interesting to further examine the integration effectiveness of Bi@SiNW structures in photovoltaics and other related optoelectronic devices. To this end, measurements of one of the most important parameters in assessing material quality—namely, minority carrier lifetime (τ)—were performed. As a matter of fact, a high τ is indicative of good material, whereas a low τ points to problems such as the presence of dislocations, impurities, and/or defects.

The measured value of τ is its effective lifetime (τ_eff_) and is considered as the average time it takes an excited minority carrier to recombine with a majority carrier. The τ_eff_ average values were extracted from the effective lifetime maps (see Supplementary Information Figure S2). We took into consideration the middle of the sample, where there is the most uniformity and homogeneity, and avoided the corners, where there are the cleavage traces. The efficiency of Bi@SiNW structures—i.e., the efficiency of the SiNW surface passivation via Bi treatment—is well quantified and evaluated by the effective surface recombination velocity (S_eff_) determination through the following expression [44,45]:

(2) $$\frac{1}{\tau_{\mathrm{eff}}}=\frac{1}{\tau_{b}}+\frac{4s_{\mathrm{eff}}}{d},$$

where τ_b_ is the bulk lifetime and d is the diameter of the nanowire (in our case, the nanowire average diameter is 200 nm and was deduced from the SEM image analysis). One can assume a high value of the bulk lifetime and then the majority of the recombination activities occur on the surface. Hence, the above equation can be written as [45]:

(3) $$S_{\mathrm{eff}}=\frac{d}{4\tau_{\mathrm{eff}}}.$$

**Figure 9:**
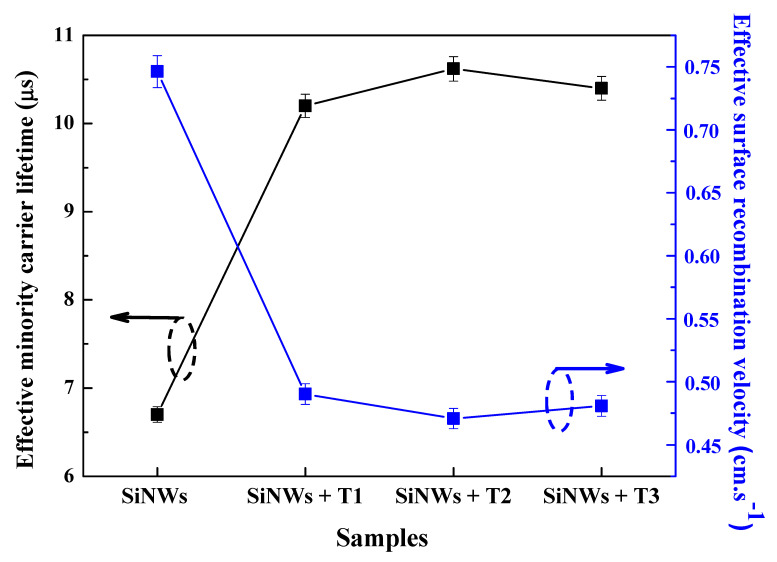
Minority carrier lifetime and surface recombination velocity of Bi-free and Bi-modified SiNW samples.

Figure 9 shows the τ_eff_ and S_eff_ values of the SiNW sample and the Bi@SiNWs samples obtained at different Bi thicknesses (T_1_, T_2_, and T_3_). One can note that the as-prepared SiNW sample exhibits a relatively short τ_eff_ and a high S_eff_ of about 6.7 µs and 0.74 cm·s^−1^, respectively. This indicates a rather low SiNWs surface quality, which is ascribed to the surface defects and dangling bands generated during the etching process. Therein lies the importance of using a powerful tool for the reduction in such recombinations—namely, surface passivation.

Indeed, Bi treatment accomplishes the purpose, since all the SiNWs samples passivated via Bi exhibit a substantial improvement in the τ_eff_ and consequently in the S_eff_, as can readily be seen from Figure 9. These enhancements are essentially due to the minimization of the dangling bond density and the diminishment of defect traps through Bi deposition and the substitution of unstable bands by more stable bands, as revealed in the FTIR analysis. Therefore, a reduction in the recombination activities occurred, and so too did surface quality enhancement. It is noteworthy that the optimal passivation of SiNWs is obtained for a moderate Bi thickness (T_2_). This is in good agreement with the PL and reflectance results on the one hand and, on the other hand, reconfirms that the passivation quality is sensitive to the amount of Bi, which requires a judicious optimization.

In a word, our findings show that Bismuth treatment plays a key role in the improvement of the SiNW surface quality. This reinforces the effectiveness of Bi as a passivation material and further proves that the Bi@SiNW composite has great potential for use in PV and optoelectronic applications.

## 4. Conclusions

SiNWs are a well-known and powerful host matrix to load and disperse a diverse set of functionalities and complexities to improve their selective properties according to the requirements. In this paper, we highlighted the effectiveness of Bi as a novel surface passivation coating for enhanced SiNW performance and for promising widespread applications. The SiNW arrays were produced through the 2-MACE method, while a simple thermal evaporation process was used to load BiNC onto the NWs. The resulting Bi@SiNW nanocomposite morphologies and compositions were studied by SEM, EDX, and FTIR spectroscopies, confirming the successful anchoring of the BiNC on the SiNW surface. A strong visible PL enhancement with a factor of nine-fold and NIR broadband emission were recorded from the Bi-modified SiNWs samples. To the best of our knowledge, this represents the first observation of NIR emission from Bi-doped SiNW arrays, and thus highlights a new family of NIR optically active Bi-doped materials. covering the entire telecommunication window. A significant suppression in the reflectance to about ~10% and ~8% is recorded for bare SiNWs and Bi@SiNWs, respectively, as compared to the ~50–90% in the untreated Si substrate. An important enhancement in the minority carrier lifetime was also obtained from the Bi@SiNW heterostructures. Several factors are behind the superior improvement of the overall properties of Bi@SiNWs and are discussed in detail. The findings strongly attest to the superior passivation of the SiNW surface via BiNC, where the Bi@SiNW heterostructure shows great promise for applications in PV, optoelectronics, telecommunication, and other fields. We anticipate that this multifunctional novel Bi@SiNWs heterostructure will be a starting point for further research.

## Figures and Tables

**Table 1:** PL spectral features of Bi-free and Bi-modified SiNWs samples.

Sample Code	Integrated PL Intensity	PL Intensity Enhancement Factor with Respect to Bare SiNWs
SiNWs	0.024	--
SiNWs + T_1_	0.167	6.95
SiNWs + T_2_	0.224	9.33
SiNWs + T_3_	0.134	5.58

## Supplementary Materials

The following are available online at https://www.mdpi.com/2079-4991/10/8/1434/s1: Figure S1: Cross-sectional view SEM image at higher magnification of the prepared SiNWs. Figure S2: Effective lifetime map and histogram of SiNWs sample.
