# Supplementary material for: Highly Efficient Silicon Nanowire Surface Passivation by Bismuth Nano-Coating for Multifunctional Bi@SiNWs Heterostructures
Source: Nanomaterials (Basel). 2020 Jul 23;10(8):1434. doi: 10.3390/nano10081434 (PMC7466647; doi:10.3390/nano10081434)
Supplement: Supplementary file 1 [file nanomaterials-10-01434-s001.pdf]

nanomaterials

Supplementary Information

Highly Efficient Silicon Nanowires Surface Passivation by Bismuth Nano-Coating for Multifunctional Bi@SiNWs Heterostructures

Mariem Naffeti [1,2,3,*], Pablo Aitor Postigo [2], Radhouane Chtourou [1] and Mohamed Ali Zaïbi [1,3]

1 Laboratory of Nanomaterials and Systems for Renewable Energies (LaNSER), Research and Technology Center of Energy, Techno-Park Borj-Cedria, Bp 95, Hammam-Lif, 2050 Tunis, Tunisia; radhouane.chtourou@crten.rnrt.tn (R.C.); ma_zaibi@yahoo.fr (M.A.Z.)
2 Instituto de Micro y Nanotecnología, IMN-CNM, CSIC (CEI UAM+CSIC) Isaac Newton, 8, E-28760 Tres Cantos, Madrid 28760, Spain; pabloaitor.postigo@imn.cnm.csic.es
3 Tunis University—National High School of Engineering of Tunis, 5 Avenue Taha Hussein, 1008 Tunis, Tunisia
* Correspondence: naffeti.mariam@gmail.com

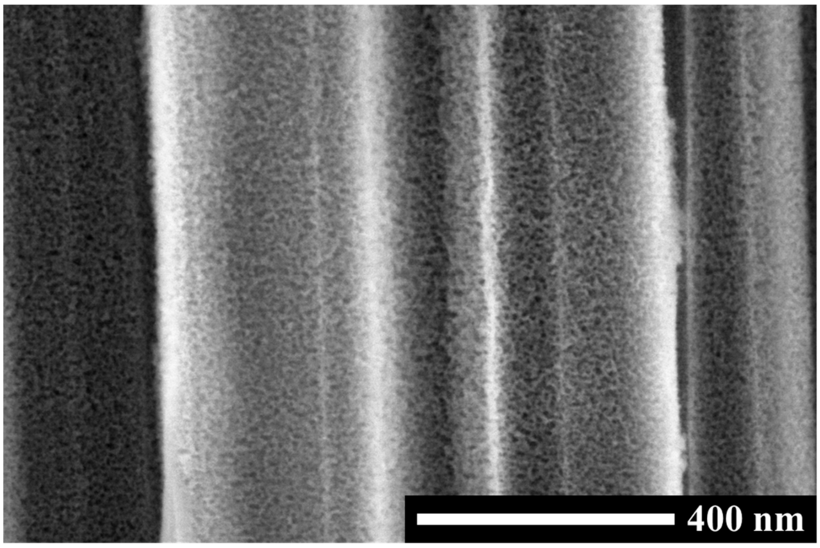

Figure S1. Cross-sectional view SEM image at higher magnification of the prepared SiNWs.

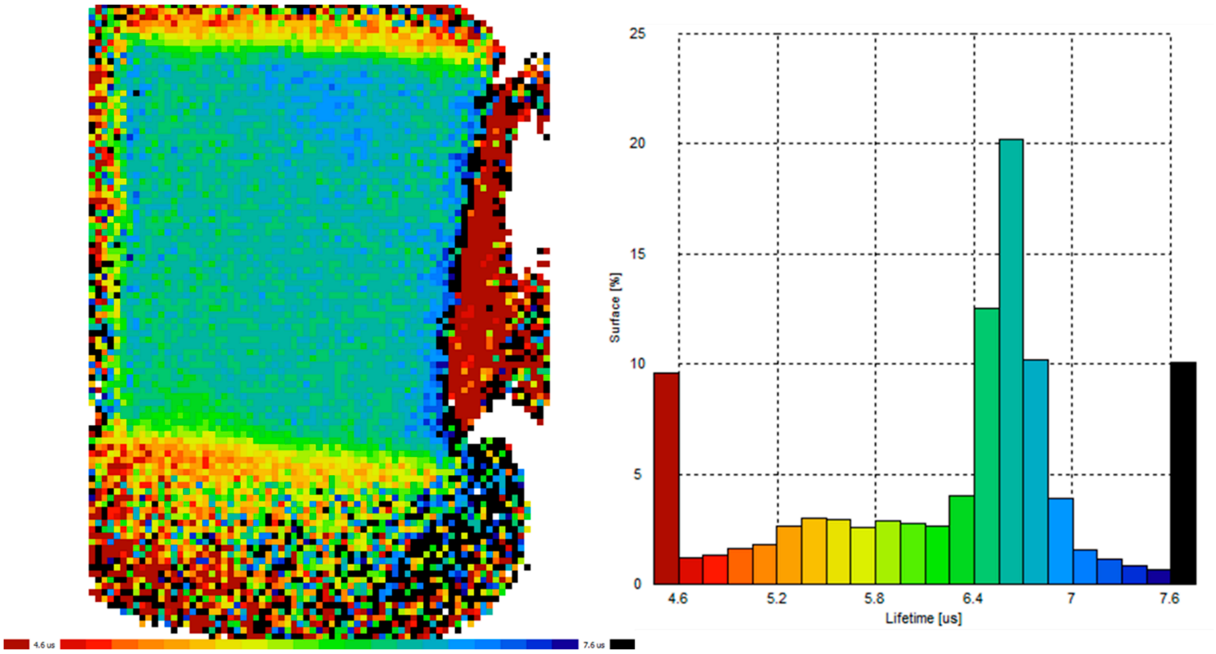

Figure S2. Effective lifetime map and histogram of SiNWs sample.
